# The natural compound obtusaquinone targets pediatric high-grade gliomas through ROS-mediated ER stress

**DOI:** 10.1093/noajnl/vdaa106

**Published:** 2020-08-27

**Authors:** Jian Teng, Ghazal Lashgari, Elie I Tabet, Bakhos A Tannous

**Affiliations:** 1 Experimental Therapeutics and Molecular Imaging Unit, Department of Neurology, Neuro-Oncology Division, Massachusetts General Hospital, Boston, Massachusetts, USA; 2 Program in Neuroscience, Harvard Medical School, Boston, Massachusetts, USA

**Keywords:** cancer stem cells, endoplasmic reticulum stress, obtusaquinone, pediatric high-grade gliomas, reactive oxygen species

## Abstract

**Background:**

Pediatric high-grade gliomas (pHGGs) are aggressive primary brain tumors with local invasive growth and poor clinical prognosis. Treatment of pHGGs is particularly challenging given the intrinsic resistance to chemotherapy, an absence of novel therapeutics, and the difficulty of drugs to reach the tumor beds. Accumulating evidence suggests that production of reactive oxygen species (ROS) and misfolded proteins, which typically leads to endoplasmic reticulum (ER) stress, is an essential mechanism in cancer cell survival.

**Methods:**

Several cell viability assays were used in 6 patient-derived pHGG cultures to evaluate the effect of the natural compound obtusaquinone (OBT) on cytotoxicity. Orthotopic mouse models were used to determine OBT effects in vivo. Immunoblotting, immunostaining, flow cytometry, and biochemical assays were used to investigate the OBT mechanism of action.

**Results:**

OBT significantly inhibited cell survival of patient-derived pHGG cells in culture. OBT inhibited tumor growth and extended survival in 2 different orthotopic xenograft models. Mechanistically, OBT induced ER stress through abnormal ROS accumulation.

**Conclusion:**

Our data demonstrate the utility and feasibility of OBT as a potential therapeutic option for improving the clinical treatment of pHGGs.

**Key Points**

- Obtusaquinone (OBT) significantly inhibits cell survival in patient-derived pediatric glioma cultures.
- OBT inhibits tumor growth and extended survival in orthotopic xenograft models.
- OBT induces endoplasmic reticulum stress through reactive oxygen species accumulation.

**Importance of the Study**

We tested the antineoplastic effect of the natural compound obtusaquinone in patient-derived pediatric high-grade glioma cell cultures and orthotopic xenograft models. We found that this agent significantly reduces cell survival in vitro, inhibits tumor growth, and leads to extended survival in vivo. Mechanistic studies suggested that obtusaquinone effect is through endoplasmic reticulum stress caused by abnormal accumulation of reactive oxygen species. These results open the door for potential evaluation of this compound for the treatment of pediatric high-grade glioma patients in the clinic.

Brain and other nervous system tumors are the leading cause of cancer death among men younger than 40 years and women younger than 20 years, accounting for 26% of childhood cancer deaths.^1^ These tumors are mostly inaccessible to chemotherapy and surgical resection. The standard of care focal radiation provides temporary improvement and stabilization of symptoms, but only extends median survival by 3 months. Refractory disease and relapses are frequent events despite intensive multimodality treatment.^2^ When compared with their adult counterparts, pediatric high-grade gliomas (pHGGs) appear in different locations of the brain, carrying diverse molecular genetics, and respond very differently to adjuvant therapies since young bodies and brains are still growing and developing. Clinical selection of effective therapeutic drugs for pHGG treatment is limited.^3^ Thus, there is an urgent unmet need to identify new targets for developing effective therapies and management strategies against pHGGs.^4^

Continuous oxidative stress resulting from the generation of reactive oxygen species (ROS) by environmental factors or cellular mitochondrial dysfunction is associated with cancer progression and metastasis.^5,6^ A moderate increase in ROS can promote cancer cell proliferation and differentiation, whereas excessive amounts of ROS can cause oxidative damage to crucial cellular macromolecules and lead to cancer cell death.^7,8^ In addition, continuous ROS generation induces accumulation of misfolded proteins in the endoplasmic reticulum (ER), leading to ER stress and apoptosis.^9,10^ Therefore, modulating ROS homeostasis or oxidative stress responses has been proposed as an effective therapeutic strategy for cancer.^11,12^ Through small molecule drug screening, we identified the natural compound obtusaquinone (OBT), which induces intracellular ROS accumulation with substantial toxicity toward different adult cancer cell lines.^13^ In this study, we aimed to evaluate the antineoplastic activity of OBT in different patient-derived pHGG cultures and in xenograft animal models and further dissected the mechanism of action of this compound in these tumors.

## Materials and Methods

### Cell Culture and Reagents

Primary pediatric glioblastoma (GBM) cells established from 2 patient tissues post-chemotherapy (SJ-GBM2) and post-chemotherapy/postmortem (CHLA-200) were obtained from the Children’s Oncology Group (Texas Tech University Health Sciences Center). These cells were grown in Dulbecco’s modified Eagle medium (Gibco) supplemented with 10% fetal bovine serum and 100 U penicillin and 0.1 mg/mL streptomycin (Sigma). Patient-derived pediatric GBM cell culture (SU-GBM2) and patient-derived diffuse intrinsic pontine glioma (DIPG) cell cultures (SU-DIPG-IV, SU-DIPG-VI, SU-DIPG-XIII) were provided and previously characterized by Dr Michelle Monje with approval of the Institutional Review Board (Stanford University).^14–16^ These cells were maintained as neurospheres in human neural stem cell media (NeuroCult; STEMCELL Technologies) supplemented with recombinant human epidermal growth factor (20 ng/mL), basic fibroblast growth factor (10 ng/mL), platelet-derived growth factor-AA (PDGF-AA, 10 ng/mL), PDGF-BB (10 ng/mL; all from Shenandoah Biotechnology, Inc.), and heparin (2 µg/mL; Sigma).

OBT was purchased from Gaia Chemicals and resuspended in dimethyl sulfoxide (DMSO) at 20 mg/mL. The antioxidants *N*-acetyl-L-cysteine (NAC) was obtained from Sigma-Aldrich. D-luciferin was purchased from Gold Biotechnology and resuspended at 25 mg/mL in PBS. Coelenterazine was purchased from NanoLight Technologies and resuspended at 10 mg/mL in acidified methanol.

Cells were transduced with a lentivirus vector expressing *Gaussia* luciferase (Gluc; for in vitro work)^17,18^ or firefly luciferase (Fluc, for in vivo work) by directly adding the lentivirus vector to these cultures at a multiplicity of infection of 10 which typically yields more than 90% transduction efficiency.^17,19^ Cell viability was measured by assaying aliquots of conditioned medium for Gluc activity using 50 µL of coelenterazine (10 µg/mL) and a luminometer. ER stress and cell apoptosis were assessed using Gluc-based assay as we previously described.^20,21^ Stem cell frequency was analyzed by sphere limiting dilution analysis and calculated using the ELDA algorithm.^22^

### In Vivo pHGG Patient-Derived Xenograft Models

All animal studies were approved by the Massachusetts General Hospital Subcommittee on Research Animal Care following guidelines set forth by the National Institutes of Health Guide for the Care and Use of Laboratory Animals. For DIPG model, around 50 000 SU-DIPG-VI cells (in small neurospheres) were injected in the pons of young (4-week-old) immunodeficient mice using the following stereotactic coordinates: 0.5 mm posterior to lambda, 1 mm lateral to the sagittal suture, and 4.5 mm deep.^16,23^ OBT was administered intraperitoneal (i.p.) at 7.5 mg/kg body weight in 20% (2-hydroxypropyl)-β-cyclodextrin (Sigma) in a total volume of 100 µL per mouse. Control groups received an equivalent amount of vehicle diluent. For GBM model, 50 000 SJ-GBM2 cells were injected in the frontal lobe of immunodeficient mice with the stereotactic coordinates: anterior–posterior +0.5 mm, mediolateral +2.0 mm, dorsoventral −2.5 mm from the bregma, as we previously described.^24^ OBT was injected intranasally at 0.75 mg/kg body weight. For both models, tumor growth was monitored over time by in vivo Fluc bioluminescence imaging after i.p. injection of D-luciferin using the Xenogen IVIS 200 Imaging System (PerkinElmer) as we previously described.^17,24^ Mice were monitored for signs of distress or systemic toxicity and weights were recorded on a weekly basis.

### Statistical Analysis

GraphPad Prism v6.01 software was used for statistical analysis of all data. A *P* value of less than .05 was considered to be statistically significant. For analysis between multiple groups, ANOVA and Tukey’s post hoc test were performed as indicated. The median inhibition concentration (IC_50_) and the half-maximal effective concentration (EC_50_) were calculated using nonlinear regression analysis. All experiments were repeated at least 3 times. Survival was analyzed using Kaplan–Meier curves and log-rank (Mantel**-**Cox) tests. All statistical tests were 2-sided. Additional methods can be found in the Supplementary Methods file.

## Results

### OBT Inhibits pHGGs Cell Proliferation

**Figure 1.**
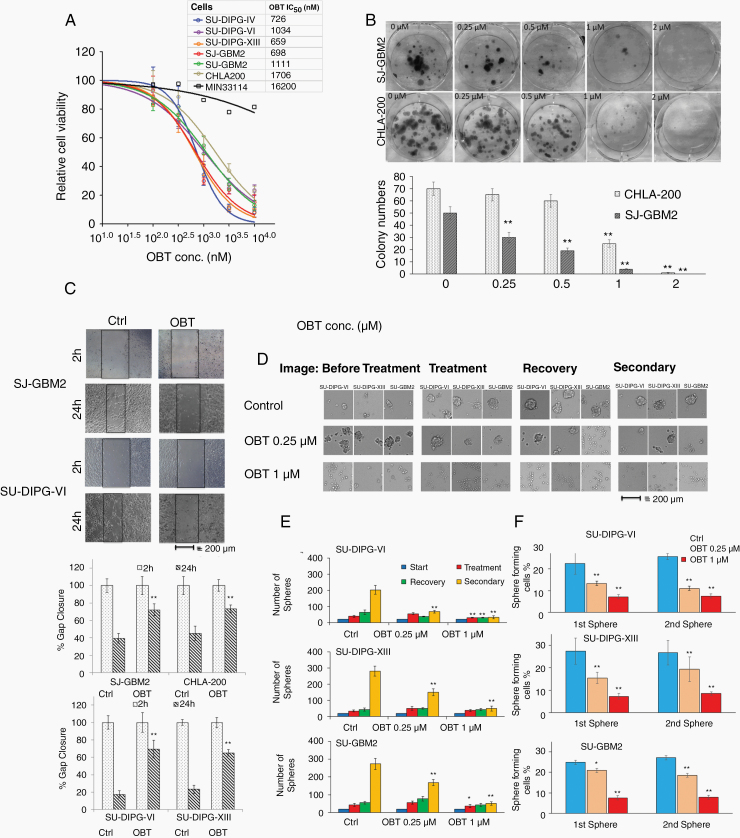
OBT inhibits pHGG cell proliferation. (A) Different patient-derived pHGG cells (SU-DIPG-IV, SU-DIPG-VI, SU-DIPG-XIII, SJ-GBM2, SU-GBM2, CHLA-200, and human fibroblast culture MIN 33114) expressing Gluc were treated with different doses of OBT for 72 h and cell viability was monitored by measuring an aliquot of conditioned medium for Gluc activity. Data presented as percentage Gluc expression in which the control untreated sample is set at 100%. OBT IC_50_ values in different cultures were calculated with Graphpad Prism and are presented on the figure. (B) Dose-dependent effect of OBT on the long-term growth of SJ-GBM2 and CHLA-200 cells as monitored by colony-forming assay. Cells at low confluency were treated with OBT for 16 h and allowed to recover in the absence of OBT for 12 days. Colonies were counted and plotted; **P < .01, by one-way analysis of variance (ANOVA). (C) Effect of OBT on pHGG cell migration. A monolayer of SJ-GBM2 or SU-DIPG-VI cell cultures was scratched using a pipette tip and treated with 0.25 µM of OBT. Cell migration was imaged at 2 and 24 h posttreatment by microscopy (40× magnification), representative images from each group are shown. Boxes define areas lacking cells; scale bar, 200 µm. Cell migration was quantified using ImageJ. **P < .01 OBT versus control at 24 h. (D and E) Effect of OBT using limiting dilution sphere-forming assay in SU-DIPG-VI, SU-DIPG-XIII, and SU-GBM2 cells. Neurospheres were treated with 0.25 µM or 1 µM OBT or vehicle control for 4 days. Spheres were then left off treatment for 5 days to allow their recovery. Recovered spheres were dissociated, and 1000 cells were plated in new 48-well plates to measure secondary sphere formation 5 days later. Representative images from each group are shown; scale bar, 200 µm (D). Total sphere numbers in the well recorded at each event; *P < .05, **P < .01 versus control by one-way ANOVA (E). (F) Sphere limiting dilution analysis comparing stem cell frequency was evaluated using the extreme limiting dilution analysis. Different numbers (1–1000) of pHGG cells were plated as single cells. The number of cells that can form a sphere was quantified at day 5 (first sphere) and day 15 (second sphere); *P < .05, **P < .01 OBT versus control by chi-square test.

Our previous work showed that OBT exhibits substantial toxicity toward different cancer cell lines including adult GBM cells. To test the effect of this compound against pediatric brain tumors, we first assembled a panel of pHGG cells consisting of 3 different patient-derived DIPG cell cultures (SU-DIPG-IV, SU-DIPG-VI, and SU-DIPG-XIII), 1 patient-derived pediatric GBM cell culture (SU-GBM2), and 2 established pediatric GBM cell lines (SJ-GBM2 and CHLA-200). We performed cell viability analysis using the naturally secreted *Gaussia* luciferase (Gluc)-based assay and compared the results to human fibroblasts MIN 33114 cells. All pHGG cells tested were sensitive to OBT with an IC_50_ ranging from 659–1706 nM across all cultures (726 nM, 1034 nM, 659 nM, 1111 nM, 694 nM, and 1706 nM, for SU-DIPG-IV, SU-DIPG-VI, SU-DIPG-XIII, SU-GBM2, SJ-GBM2, and CHLA-200, respectively), compared to an IC_50_ of 16 µM for human fibroblasts MIN 33114 (Figure 1A). The OBT effect on pHGG cells was confirmed using the colony-forming assay. A 16-h treatment with 0.25 µM OBT significantly inhibited colony formation and 2 µM OBT was sufficient to totally abrogate colony formation in SJ-GBM2 and CHLA-200 cells (Figure 1B).

To evaluate whether OBT could regulate the motility of these cells, we performed a scratch assay on a monolayer of SJ-GBM2 cells and SU-DIPG-VI (plated in the presence of Synthemax II-SC which yields a monolayer culture). Twenty-four hours post-scratch, cells treated with 0.1% DMSO had the ability to move and fill large parts of the scratch. However, in both cultures, cells treated with 0.25 µM OBT showed a significant decrease in cell motility compared to control (*P* < .01; Figure 1C). Furthermore, neurospheres formation, growth/recovery, and secondary sphere formation (by dissociating spheres posttreatment and re-plating single cells in a new well) were monitored in cultures of SU-DIPG-VI, SU-DIPG-XIII, and SU-GBM2 stem-like cells. OBT treatment resulted in the dissociation of neurospheres into single cells and caused extensive cell death. About 0.25 µM or 1 µM OBT yielded a significant inhibition in sphere numbers in all 4 cultures (*P* < .05; OBT vs control). The secondary sphere formation responded to OBT treatment more profoundly in all cultures (*P* < .01; Figure 1D and E). These results were confirmed using sphere limiting dilution assay to compare the in vitro self-renewal ability of pHGG stem-like cells. About 0.25 µM or 1 µM OBT caused a remarkable decrease in the proportion of cells that can form new spheres (*P* < .05, OBT vs control; Figure 1F). These results demonstrate that OBT exerted a marked cytotoxic effect on pHGG cells by inhibiting stem cell renewal and cell proliferation.

In contrast to adult GBMs where temozolomide has a clear therapeutic benefit, within the pHGG patient populations, temozolomide regimens on its own had failed to improve outcomes in children with HGGs.^3^ We thought to evaluate if OBT may sensitize pHGGs to this chemotherapeutic agent. OBT on its own had significantly increased killing effects in all tested cultures; however, we did not observe any additive effect with temozolomide (Supplementary Figure 1).

### In Vivo Effect of OBT on Patient-Derived pHGG Xenograft Models

**Figure 2.**
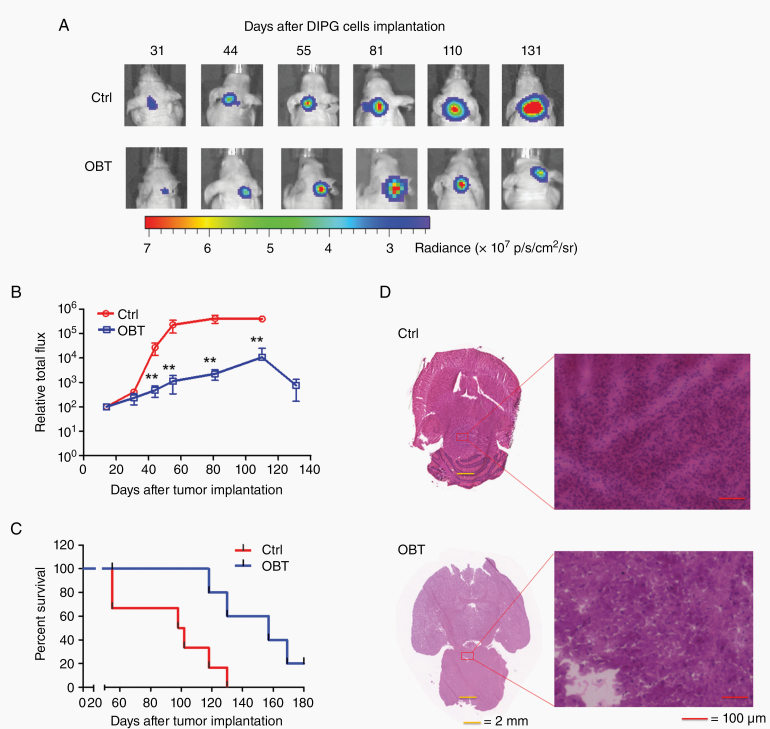
OBT inhibits tumor growth and increases mouse survival in the pHGG patient-derived xenograft mouse model. SU-DIPG-VI cells expressing Fluc were injected in the pons of young (4 weeks old) immunodeficient mice. Twenty days post-implantation, animals were randomized into 2 groups and intraperitoneally injected once a day, 3 days per week for 4 weeks, with OBT (7.5 mg/kg body weight) or vehicle control (Ctrl; *n* = 8–10 per group). Mice were imaged weekly and survival was recorded. (A) Representative images of one mouse from each group are shown over time. (B) Quantification of tumor-associated Fluc radiance intensity presented as photons/sec/cm^2^/surface radiance; data presented as mean ± SD, *n* = 8–10; **P < .01 OBT versus control by ANOVA and Tukey’s post hoc test. (C) Kaplan–Meier survival curve with *P* = .0091(2-sided log-rank test; *n* = 8–10). (D) On day 21 after implantation, 2 mice from each group were sacrificed, brains isolated, sectioned, and analyzed by hematoxylin and eosin staining. Micrographs from one representative mouse per group are shown. Left, horizontal sections at the largest cross-sectional area of formalin-fixed paraffin-embedded whole brains; scale bar, 2 mm. Right, the center of the tumor is shown at higher magnification (×200); scale bar, 100 µm.

Given our promising results obtained in different cell cultures, we evaluated the effect of OBT against pHGGs orthotropic xenograft mouse models. Around 50 000 SU-DIPG-VI cells expressing Fluc (in small neurospheres) were implanted intracranially into the pons of young (4 weeks old) immunodeficient mice. Animals were imaged once a week to follow tumor progression using Fluc bioluminescence imaging. Twenty-days post-implantation, when tumors were formed, OBT (7.5 mg/kg body weight) was intraperitoneally injected once a day, 3 days per week over 4 weeks, and the drug vehicle as a control (*n* = 8–10/group). OBT-treated group showed a statistically significant decrease in tumor growth compare to the control group (Figure 2A). At day 55 post-implantation, the average Fluc signal from brain tumors was 110 times higher in the control group versus OBT (*P* < .001; Figure 2B). In addition, OBT-treated group showed a statistically significant increase in lifespan, with a median survival of 157 days compared to 100 days for the control group (median survival ratio OBT vs control: 1.6, 95% CI of ratio = 1.14–1.95; *P* = .0091; Figure 2C). OBT-treated mice remained healthy with no sign of toxicity throughout the treatment. The difference in tumor growth between the 2 groups was further confirmed by hematoxylin and eosin staining of brain sections where a clear difference in tumor size was observed (Figure 2D).

Since previously we have developed an intranasal delivery method to bypass the limiting properties of the blood–brain barrier,^25^ we tested intranasally administration of OBT in another in vivo model. This method allows us to achieve an optimal antitumor effect, lower the therapeutic doses of the compound, and eliminate possible side effects. Interestingly, in SJ-GBM2 xenograft mouse model, after treatment with OBT (0.75 mg/kg body weight, 3 days per week for 2 weeks), quantitation of tumor signal revealed significant inhibition of tumor growth (*P* < .001; Supplementary Figure 2A and B), which correlated with increased mouse survival where OBT-treated cohort had a median survival of 102 days versus 41.5 days for the control group (*P* = .0035; Supplementary Figure 2C). Together, these results indicate that treatment with OBT potently suppresses pHGG tumor growth in different areas of the brain and using a different delivery route with no apparent toxicity in mice.

### OBT Activates ROS-Dependent ER Stress

**Figure 3.**
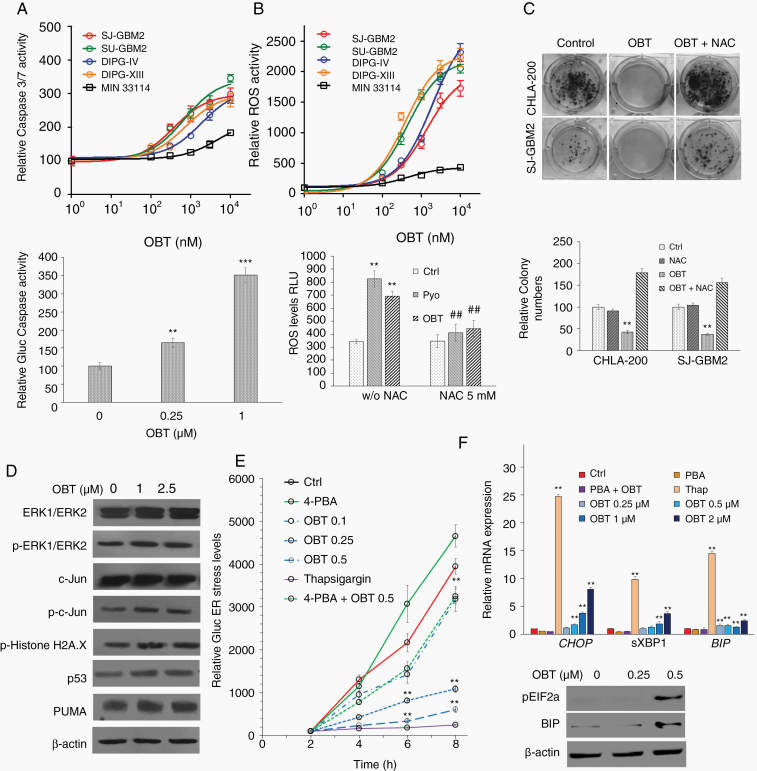
OBT induces ROS, apoptosis, and ER stress in pHGGs. (A) Upper: Caspase 3/7 activity measured 16 h posttreatment with 100 nM–10 µM OBT in SJ-GBM2, SU-GBM2, SU-DIPG-VI, SU-DIPG-XIII, and human fibroblasts MIN 33114 cells. Data are normalized to vehicle control for each culture. The average of experimental triplicates (±SD) is shown. Lower: SJ-GBM2-expressing GFP-DEVD-ssGluc apoptosis sensor was treated with different doses of OBT and the level of Gluc in conditioned medium was analyzed 24 h later (**P < .01, ***P < .001 vs control). (B) Upper: Reactive oxygen species (ROS) were measured 4 h after treatment with 100 nM–10 µM OBT in SJ-GBM2, SU-GBM2, SU-DIPG-VI, SU-DIPG-XIII, and human fibroblasts MIN 33114 cells; lower: SJ-GBM2 cells were treated with 0.1% DMSO, 1 M pyocyanin (Pyo), or 0.5 µM OBT in the presence or absence of NAC (5 mM) and ROS level was measured 4 h later. Data are presented as the mean of fluorescence intensity ± SD, **P < .01 versus control, ##*P* < .01 NAC treatment versus no NAC. (C) SJ-GBM2 and CHLA-200 cells at low confluency were treated with OBT in the presence or absence of NAC for 16 h and allowed to recover in growth medium in the absence of OBT for 12 days. Colonies were counted and plotted; **P < .01versus control by one-way ANOVA. (D) SJ-GBM2 cells were treated for 24 h with 1–2.5 µM OBT and cell lysates were analyzed by Western blotting for ERK1/2 phosphorylation (p-ERK1/2) and ERK1/ERK2, c-jun phosphorylation (p-c-jun) and c-jun, as well as phosphor-Histone H2AX, p53, and p53 upregulated modulator of apoptosis (PUMA), and β-actin for normalization of protein loading. (E) Effect of OBT on ER stress. SJ-GBM2 cells expressing the secreted Gluc were treated with different doses of OBT, thapsigargin (Thap, 100 nM) as a positive control, with and without the ER stress inhibitor 4-phenylbutyric acid (4-PBA, 10 mM). Gluc secretion into conditioned medium was monitored over time (**P < .01 vs control). (F) Upper: mRNA from SJ-GBM2 cells treated with different doses of OBT, Thap with or without 4-PBA were analyzed by qRT-PCR for different ER stress markers including *CHOP*, *sXBP1*, and *BIP* (**P < .01 vs control). Lower: Cell lysates were analyzed by Western blotting for pEIF2a, BIP, and β-actin.

Our data revealed that OBT has a clear anti-pHGG response in both 2D and 3D culture models independent of the environmental oxygenation conditions. However, it remained elusive as to how this drug could elicit this response. We have previously shown that OBT targets and kills cancer cells by oxidative stress. We therefore evaluated if a similar pathway is activated in pHGGs. We treated 4 different pHGG cultures with a range of 100 nM–10 µM OBT for 16 h and found that this drug significantly stimulated caspase 3/7 with an EC_50_ for SJ-GBM2, SU-GBM2, SU-DIPG-VI, and SU-DIPG-XIII of 368 nM, 697 nM, 1852 nM, and 723 nM, respectively; the effect of OBT in fibroblasts was much lower with an EC_50_ of 4.9 µM (Figure 3A). This caspase activation was confirmed by another assay where OBT was tested against SJ-GBM2 cells engineered to express a fusion protein consisting of GFP-DEVD-ssGluc developed in our laboratory.^26^ Upon OBT treatment, DEVD was cleaved freeing Gluc to enter the ER and secrete into the conditioned medium in a dose-dependent manner (Figure 3A).

We next determined the effect of OBT on cellular ROS levels using a redox-sensitive fluorescent probe. We observed a dose-dependent increase in ROS levels in pHGG cells treated with OBT for 4 h with an EC_50_ for SU-DIPG-IV, SU-DIPG-VI, SU-DIPG-XIII, and SU-GBM2 to be 1367 nM, 441 nM, 1894 nM, and 374 nM, respectively, in contrast to fibroblasts which showed a minimal amount of ROS (EC_50_ = 4.1 µM; Figure 3B). As a positive control, we used the redox-active compound pyocyanin (Pyo), which also induced ROS production in SJ-GBM2 cells. Importantly, when cells were co-treated with the antioxidant NAC, a potent ROS scavenger, ROS accumulation caused by OBT or Pyo was almost totally reversed (Figure 3B). Furthermore, NAC leads to almost complete protection of OBT-induced pHGG cell death as monitored by colony formation assay (Figure 3C). These data suggest that OBT induces apoptosis in pHGGs by interfering with the cell redox state and induction of ROS.

Consistent with our previous findings in other cancer cells, Western blot analysis showed that OBT promoted downstream activation of ERK pathway, typically activated by oxidative stress, with an increase in phosphorylation of Erk1/2 and c-Jun N-terminal kinase/stress-activated protein kinase (Figure 3D). The tumor suppressor p53 is a key sensor and regulator of cellular damage and apoptosis. An increase in p53 expression and one of its downstream proapoptotic targets, p53-upregulated modulator of apoptosis (PUMA), was also detected in response to OBT treatment (Figure 3D). DNA double-strand breaks, as determined by the phosphorylation of histone 2AX (γ-H2AX), were increased in response to OBT.

Recent studies have shown that the production of ROS and ER stress are closely linked events. ROS accumulation and redox status perturbation disrupt protein folding in the ER, causing ER stress. Therefore, we evaluated whether OBT-induced ROS-dependent apoptosis is associated with ER stress. We first used an assay which we have developed to monitor ER stress based on the naturally secreted Gluc. Treatment of SJ-GBM2 cells with OBT leads to a dose- and time-dependent increase in ER stress response as measured by the immediate inhibition of Gluc processing through the secretory pathway and secretion to the conditioned medium (Figure 3E). In the same assay, thapsigargin (100 nM), one of the most commonly used inducers of ER stress, increased Gluc secretion in a similar manner. On the other hand, when SJ-GBM2 cells were pretreated with 4-phenylbutyric acid (4-PBA), a popular compound that interacts with unfolded or misfolded proteins to alleviate ER stress, Gluc secretion was restored to the same levels as the control (Figure 3E). When ER stress-related genes were considered, OBT treatment caused a dose-dependent increase in the expression of CCAAT/enhancer-binding protein homologous protein (*CHOP*), the spliced form of X-box-binding protein-1 (*sXBP1*), and the binding of immunoglobulin protein (*BIP*) mRNA levels (analyzed by quantitative real-time PCR) in SJ-GBM2 and 3 different DIPG cultures (Figure 3F; Supplementary Figure 3). Similarly, thapsigargin also stimulated *CHOP*, *sXBP1*, and *BIP* level, but pretreatment with 4-PBA (1 mM) reversed the OBT effect (Figure 3F). These results were also confirmed by examining 2 ER stress-related proteins by Western blotting, phosphorylated protein kinase RNA-like eukaryotic initiation factor 2α (p-EIF2α) and BIP, which were both elevated in response to OBT (Figure 3F). These findings indicate that ROS induction mediates OBT-induced ER stress leading to apoptosis in pHGG cells.

## Discussion

Pediatric gliomas have a remarkably different molecular and genetic profile compared to their adult counterparts; therefore, existing data on the treatment and management of adult HGGs cannot be extrapolated to pediatric gliomas. The mainstay of treatment in pHGGs is total surgical resection if clinically feasible. Several efforts have been made to decipher the biological and molecular properties of pediatric HGGs during the last 2 decades, through which new management plans and treatment approaches can be tailored to fit each tumor type. Tumors in general present high levels of oxidative stress, caused by an increase in ROS production or decrease in intracellular ROS-scavengers, believed to promote cell survival and confer resistance to therapy; however, a further increase in oxidative stress can overwhelm this stress response in tumor cells leading to cell death. We have previously shown that the natural compound OBT has antineoplastic properties against different cancer types, including adult GBM and breast cancer. In this study, we evaluated whether this natural compound has an effect against malignant pediatric gliomas. We confirmed the anticancer effects of OBT on several patient-derived pHGG cultures by implementing different cell viability, glioma stem cell self-renewal, and colony formation assays. We demonstrated that the cytotoxic effect of OBT is at least partially related to ER stress induced by increased ROS. In vivo, OBT markedly reduced tumor growth, leading to a significant increase in survival.

Our studies suggest that OBT induces cell apoptosis through oxidative stress leading to increased production of ROS, and this effect is reversed by the antioxidant NAC. The increased dependency of cancer cells on the ROS stress response pathway may be the basis for the selectivity of OBT-induced apoptosis in pHGGs. This differential response suggests that OBT targets a dependency associated with ROS homeostasis. Normal cells have low basal levels of ROS and therefore a diminished reliance on the ROS stress response pathway, whereas cancer cells have high levels of ROS and might therefore be expected to have a strong reliance on this pathway. Production of ROS is linked with the accumulation of unfolded or misfolded proteins in the ER, leading to ER stress. To lessen such circumstances, cells activate a homeostatic intracellular signaling network, called the unfolded protein response, which leads to a temporary inhibition of protein synthesis and increased expression of ER chaperone proteins. If cells fail to cope with this stress, they activate apoptosis. Our cell-based experiments suggest that OBT treatment selectively promotes ROS and apoptosis in pHGGs through ER stress. This response is coupled with oncogene-dependent oxidative stress by stabilizing P53 along with a functional increase in one of its downstream targets, proapoptotic gene PUMA. In addition, OBT induced strong activation of 2 components of the mitogen-activated protein kinase, ERK1/2 and c-jun. Activation of these signaling pathways is most likely due to a sharp increase in ROS levels. Furthermore, we confirmed that ROS activation and ER stress are the main cause of OBT-induced apoptosis in pHGGs since cotreatment with an antioxidant or compound that interacts with misfolded proteins to alleviate ER stress leads to almost a complete protection against cell death. However, it is possible that other mechanisms are also involved in the observed OBT effect on pHGGs. Recently, we showed that OBT binds to cysteine residues in Keap1, a member of the CUL3 ubiquitin ligase complex, resulting in its ubiquitination and proteasomal degradation followed by downstream activation of the Nrf2 pathway.^27^ We would expect that the same mechanism to also be involved in pHGGs and it would be interesting to evaluate KEAP1/Nrf2 pathway in future studies. Finally, we also developed an OBT analog with improved pharmacological properties, laying the path for clinical translation.^27^

## Conclusions

OBT significantly inhibits cell proliferation and survival of patient-derived pHGG cells in vitro as well as tumor growth in vivo, leading to extended survival in 2 different orthotopic xenograft models. Mechanistically, OBT induces ER stress through abnormal ROS accumulation, leading to caspase activation and cell death. Our data demonstrate the utility and feasibility of OBT as a potential therapeutic option for improving the clinical outcome of pediatric patients with HGGs.

## Supplementary Material

vdaa106_suppl_Supplementary_Material

## Notes

**Authorship statement**. Experimental design: J.T., G.L., E.T., and B.T.; implementation: J.T., G.L., and E.T.; data analysis and interpretation: J.T., G.L., and B.T.; writing: J.T., G.L., and B.T., with input and approval from all authors.
