# Supplementary material for: The natural compound obtusaquinone targets pediatric high-grade gliomas through ROS-mediated ER stress
Source: Neurooncol Adv. 2020 Aug 27;2(1):vdaa106. doi: 10.1093/noajnl/vdaa106 (PMC7592425; doi:10.1093/noajnl/vdaa106)
Supplement: vdaa106_suppl_Supplementary_Material [file vdaa106_suppl_supplementary_material.pdf]

Supplementary Methods

Lentivirus vector stable expression. The Gluc cDNA and the GFP expression cassette separated by an internal ribosomal entry site (IRES) element were cloned into a lentivirus vector under the control of the strong constitutive cytomegalovirus (CMV) promoter. Similar vector has been generated to express Fluc and mCherry fluorescent protein. To generate stable expression, different cell types were infected with these lentivirus vector at a multiplicity of infection of 10-50 transducing units per cell by adding the vector directly to the culture medium in the presence of 8 µg/ml polybrene, leading to >95% transduction efficiency in GBM cells in monolayer and neural spheres.

Sphere formation and Limiting dilution analysis assay. Patient-derived GBM stem-like cells were grown as neural spheres in stem cell medium (neurobasal medium supplemented with EGF and FGF (20 ng/mL), heparin (1:1000), B27 supplement (1:50). Spheres were then dissociated into single cells with PBS-EDTA and 1000 cells/well were seeded to image and count the secondary sphere formation five days later. Cells were plated in 96-well plates at a density of 3000-5000 cells/well. To monitor cell growth, aliquots of cell-free conditioned medium (10µL) were transferred to a white 96-well plate. Gluc activity was determined using FlexStation3 microplate reader (Molecular Device, Sunnyvale, CA) under “Flex” mode after dispensing 100µL 40 µM of coelenterazine (Nanolight, Pinetop, AZ), dissolved in acidified methanol and further diluted in phosphate buffered saline (PBS) before use.

Stem cell frequency was compared with sphere limiting dilution analysis and calculated using the ELDA algorithm (http://bioinf.wehi.edu.au/software/ elda/). Briefly, cells were grown in stem cell medium at a density range from 1 to 1000 cells/well in a 48 well plate. After 5 days of incubation, wells containing one or more cell clumps of 50-250 µm were counted as 1st spheres. These spheres were dissociated at day 10 and 2nd spheres were counted after another 5 days of incubation (day 15). Neurospheres were dissociated into single cells and 1000 cells/well in 500 µl stem cell medium were plated in 48-well ultra-low adherent culture plates. After one day, cells were treated and 4 days post-treatment, phase-contrast images were obtained to visualize the morphology of sphere. Spheres were then dissociated into single cells with PBS-EDTA and 1000 cells/well were seeded to image and count the secondary sphere formation five days later.

Gluc cell viability assay. pHGG cells engineered to express Gluc-GFP were plated in 96-well plates at a density of 3000-3000 cells/well. To monitor cell growth, aliquots of cell-free conditioned medium (10µL) were transferred to a white 96-well plate. Gluc activity was determined using FlexStation3 microplate reader (Molecular Device, Sunnyvale, CA) under “Flex” mode after dispensing 100µL 40 µM of coelenterazine (Nanolight, Pinetop, AZ), dissolved in acidified methanol and further diluted in phosphate buffered saline (PBS) before use.

Gluc cell apoptosis assay. pHGG cells engineered to express Gluc-GFP our established apoptosis reporter GFP-DEVD-ssGluc were plated in 96-well plates at a density of 3000cells/well. Induced caspase activation, leading to DEVD cleavage, freeing Gluc which is then secreted to the conditioned medium. For determination of Gluc activity in culture, 10 µl aliquots of the cell-free conditioned medium were collected in triplicates at different time points and transferred into a white 96-well plate. The Gluc activity at different time points was compared to basal level at time zero.

Scratch assay. Cell migration was studied according to the method described by Liang et al. Briefly, a monolayer of GBM cells and neurospheres (cultured in a 12-well plate in the presence of 1 µg/ml Synthemax® II-SC-substrate to attach the GBM stem-like cells; Corning, Corning, NY) was scraped in a straight line using a p200 pipette tip. Cell debris was removed by washing the

cells with culture medium, followed by treatment with TMZ and/or HU. An image from each well was acquired using phase-contrast microscope at different time points. ImageJ software (National Institute of Health, Bethesda, MD, USA) was used to record the coordinates for each scratch location using a computer-controlled stage and the mean scratch width at 24 h was calculated to the original scratch width (2 h). Each experiment was repeated three times.

Bioluminescence Imaging. The system is composed of an imaging chamber, gas anesthesia system which is connected to an oxygen cylinder and isoflurane tank, and a highly sensitive cryogenically cooled charge-coupled device camera. Fresh luciferin solution is prepared by dissolving luciferin powder (25 mg/mL) in phosphate-buffered saline. Mice were injected intraperitoneally with 150 µg (200 mg/kg) of D-luciferin substrate (Gold Biotech, St. Louis, MO) per gram body weight and transferred into the image chamber. Imaging was acquired 10 min post-luciferin injection and the image inte nsity was quantitated using the Living Image software 3.0 from Xenogen Imaging Technologies (Perkin-Elmer, Waltham, MA).

Two weeks after tumor transplantation, mice were sacrificed by transcardial perfusion. They were injected with phosphate buffered saline (PBS) followed by 4% paraformaldehyde (PFA), under deep anesthesia with intraperitoneal (i.p) injection of ketamine (100 mg/kg) and xylazine (5 mg/kg). Tumors were collected, soaked in 30% sucrose overnight and sectioned into 7 **µ**m sections. Sections were mounted on slides and stained with Hematoxylin and Eosin and analyzed by microscopy.

Apo-ONE® Homogeneous Caspase-3/7 Assay. The induction of apoptosis by the most active extracts from each plant was evaluated by measuring the caspase 3/7 activity on different cancer cell lines with the Caspase-Glo® 3/7 assay kit (Promega). All four cancer cell lines were seeded at a density of 104 cells per well on 96-well microtitre plates, and were allowed to adhere overnight. These cells were treated with the extracts at different concentrations (½ × IC50, IC50 and 2 × IC50) or DMSO (0.5%) as negative control, and the plates were incubated at 37 °C with 5% CO2 for 24 h. After treatment, the Caspase-Glo® 3/7 was prepared according to manufacturer's guidelines, and 100 µL of the reagent was added per well and incubated for 1 h at room temperature in the dark. Following this incubation, the luminescence was measured on a microplate reader (Synergy Multi-Mode Reader, BioTek). The data was analysed, and expressed as percentage of the untreated cells (control) and fold change. The Caspase-Glo 3/7 assay reagent (Promega, Madison, WI) was used for caspase detection in treated cells in vitro. The reagent provides a proluminescent caspase-3/7 substrate, which contains the tetrapeptide sequence DEVD, in combination with luciferase and a cell-lysing agent. The addition of the Caspase-Glo 3/7 reagent directly to the assay well results in cell lysis, followed by caspase cleavage of the DEVD substrate, and the generation of luminescence. The amount of luminescence as displayed on the readout is proportional to the amount of caspase activity in the sample. The Caspase-Glo 3/7 (Promega Corporation, Madison, WI) lyophilized substrate (DEVD-aminoluciferin powder) was dissolved in Caspase-Glo 3/7 buffer (lysis buffer) as recommended by Promega, or, alternatively, was dissolved in an equal volume of Dulbecco's PBS. In order to observe the effects of DEVD-aminoluciferin alone and inactivate luciferases saturated in the DEVD-aminoluciferin powder, DEVD-aminoluciferin powder dissolved in PBS was heated at 85˚C for 20 min, and then cooled in a 37˚C water bath for 20 min before addition to cells (to prevent heat damage to cells).

Western blotting assay . Cells were cultured in 6-well plates for 24 hours. Cells were then treated with 0, 0.1, 0.25, or 0.5µM OBT for 24 hours. Total protein was harvested using RIPA buffer (89900, Thermo-Fisher) and 1× protease inhibitor cocktail (78440 Thermo-Fisher). 30 µg of protein was then loaded for electrophoresis on 4-12% tris glycine gel (XP04120BOX Invitrogen)

using tris glycine running buffer (BP-150 Boston Bioproducts), and then transferred to nitrocellulose membranes using tris glycine transfer buffer (LC3675 Invitrogen). Membranes were incubated overnight with primary antibodies at 1:1000 dilutions in 5% nonfat milk powder in PBS/0.1%TWEEN-20. Dilutions were adjusted to obtain optimal results. The primary antibodies used were: phosphor-EIF2α (3957S Cell Signaling), BIP (3177 Cell Signaling), β-actin (3700 Cell Signaling). Membranes were then probed with secondary antibody conjugated to horseradish peroxidase (HRP) for 1 hour at room temperature. The secondary antibodies used were: anti-rabbit IgG (7074 Cell Signaling), anti-mouse IgG (7076 Cell Signaling). Proteins were visualized with SuperSignal West Pico Plus Chemiluminescent Substrate (34580 Thermo-Fisher).

RNA isolation and quantitative real-time PCR. Cells from 6 well plates were collected, and RNA was isolated using RNeasy mini kit (Qiagen). RNA concentration was determined using a Nano-Drop spectrophotometer. Then cDNA was synthesized using 5X All in one RT MasterMix kit (G490 abm). Different genes expression was analyzed by quantitative real-time PCR using an ABI 7500 sequence detection system thermal cycler (Applied Biosystems). The expression of different human genes was analyzed in this study including: CHOP, sXBP1, Grp78 (BIP), and GADPH as housekeeping gene. The primers sequences are as follow: GAPDH: Forward 5'-ACAACTTTGGTATCGTGGAAGG-3' and Reverse 5'- GCCATCACGCCACAGTTTC-3'; CHOP: Forward 5'-GGAAACAGAGTGGTCATTCCC-3' and Reverse 5'-CTGCTTGAGCCGTTCATTCTC-3'; sXBP1: Forward 5'-GGTCTGCTGAGTCCGCAGCAGG-3' and Reverse 5'-GGGCTTGGTATATATGTGG-3'; GRP78: Forward 5'-CATCACGCCGTCCTATGTCG-3' and Reverse 5'-CGTCAAAGACCGTGTTCTCG-3'.

Clonogenic assays . Tumor cells were plated in a 6-well plate at a concentration of 400 cells/well in regular growth media 200-500 cells per well. Cells were treated with control, OBT (3 mM) or OBT (3 mM) + NAC (3 mM) for duration of 16 to 18 hours. After treatment cells were allowed to grow for 14 days, with media replenishment occurring after 7 days. At the end, gently remove all medium from the dish. Carefully add sufficient precooled methanol 100% methanol to cover the cells. Cover the dish and incubate at room temperature for 20 min. Remove the methanol and rinse the cells with tap water. Cells were then fixed with and stained with 0.1% Crystal Violet at room temperature. Stain was washed off three times with tap water after 5 min incubation. Dishes were photographed and colonies of >50 cells were manually counted.

ROS measurement and Superoxide Staining. ROS-ID Total ROS detection kit was from Enzo (Farmingdale, NY, USA). Tumor cells were plated on Cultrex-coated coverslips (thin layer only) in a 24-well plate at a density of 50,000/coverslip. Cells were given several hours to attach to coverslips with proper positive and negative controls. Cells were incubated for a total of 48 hours prior to imaging. The night before imaging, half of the wells had 5mM NAC added to inhibit ROS. The day of imaging, wells were changed to fresh media. Negative control coverslip was treated with 5mM NAC for 30 minutes prior to induction. Cells were then incubated for an hour at 37°C with the induction solution - 2x ROS Detection Solution in phenol red-free media at a final concentration of 2.5μM each. For positive control, pyocyanin, a ROS inducer, was added to one of the coverslips. The coverslips were washed 2x with provided wash buffer, and images were captured with a Zeiss LSM 780 confocal microscope (Carl Zeiss AG, Göttingen, Germany) using a 40 × immersion lens. Oxidative stress was captured using excitation of 490nm and emission of 525; Superoxide detection was captured using excitation of 550nm and emission of 620nm.

Supplementary Figure 1

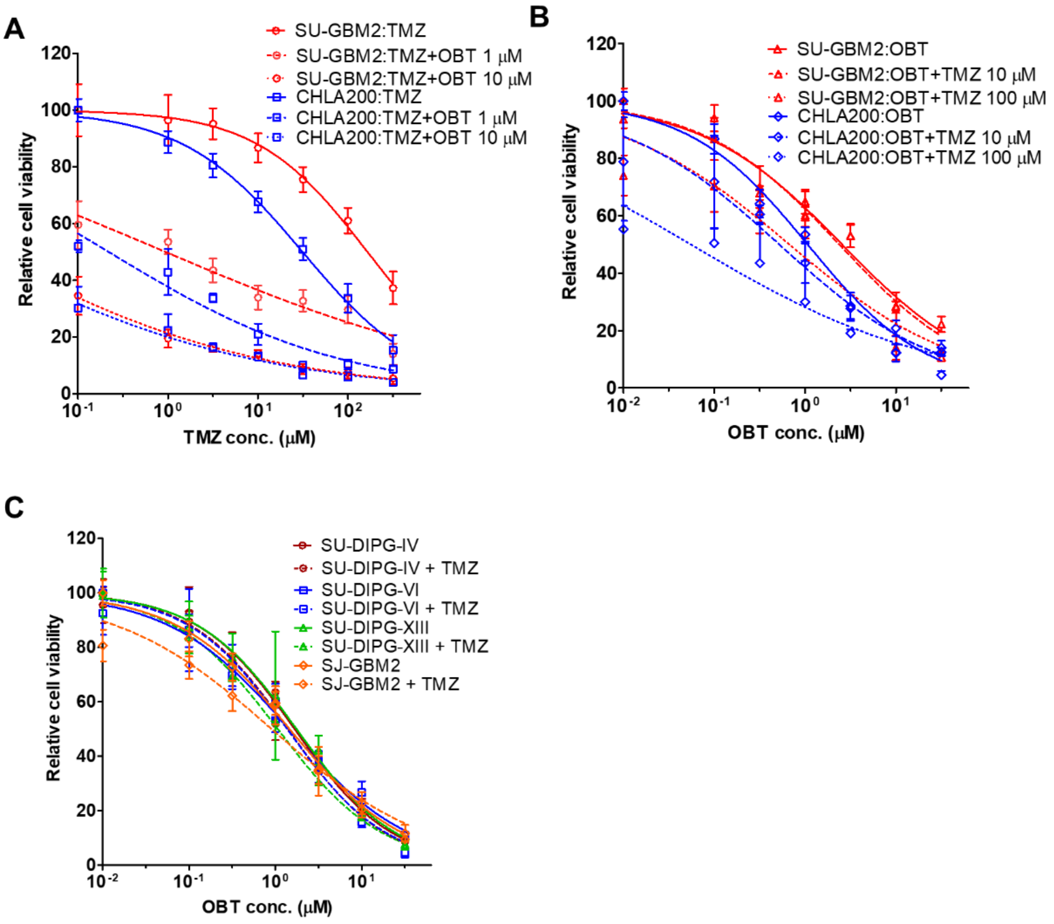

Fig. S1. Combination of OBT with temozolomide in phGG cultures. (A) SU-GBM2 and CHLA-200 cells were treated with escalating concentrations of TMZ (0.1 -300 μM) in the absence or presence of 1 or 10 μM OBT. (B) SU-GBM2, and CHLA-200 cells were treated with escalating concentrations of OBT (0.01 -30 μM) in the absence or presence of 10 or 100 μM TMZ. (C) DIPG-IV, DIPG-VI, and SJ-GBM2 cells were treated with escalating concentrations of OBT (0.01 -30 μM) in the absence or presence of 100 μM TMZ. For (A-C), cell viability was measured three days later using the Gluc assay and normalized to the vehicle control (0.1% DMSO), which was set at 100%. Results are shown as the mean ± SD of 8 wells.

Supplementary Figure 2

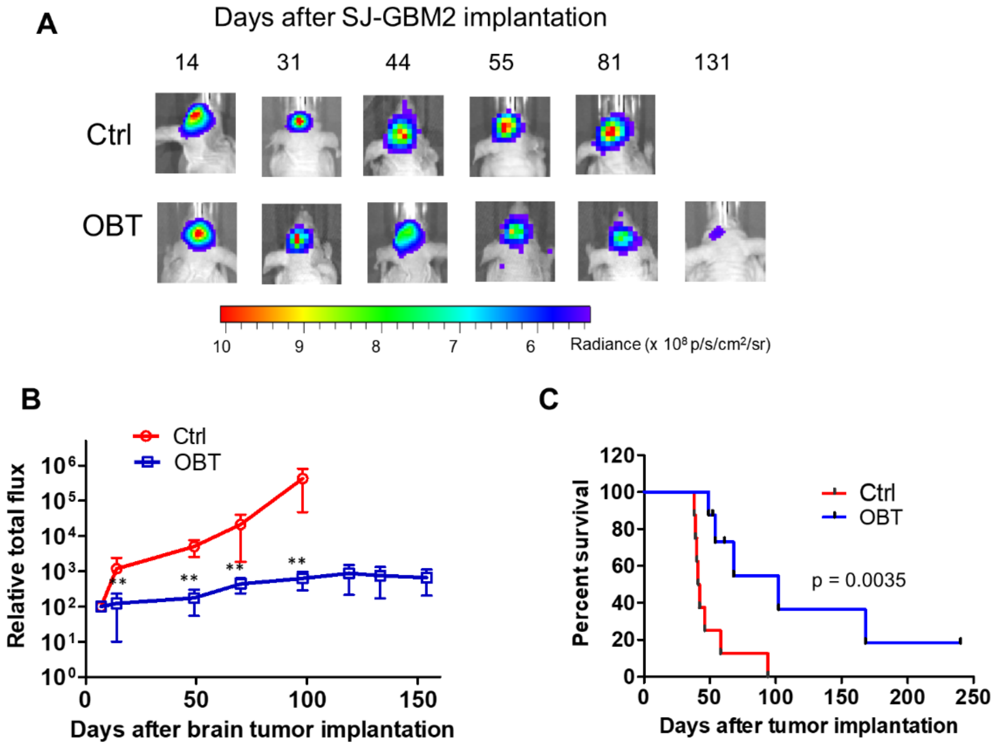

Fig. S2. Effect of OBT on orthotopic pHGG mouse model *in vivo*. SJ-GBM2 cells expressing Fluc were injected intracranially in 6 weeks-old immunodeficient mice. 14 days later, animals were randomized into two groups and intranasally injected once a day, 3 days per week for 4 weeks with OBT (0.75mg/kg body weight) or vehicle control (Ctrl) (n = 8-9 per group). Mice were imaged weekly and survival was recorded. (A) Representative images of one mouse from each group is shown over time. (B) Quantification of tumor-associated Fluc radiance intensity presented as photons/sec/cm2/surface radiance; Data presented as mean ± SD; ***p*<0.01 OBT vs. control (n=8-9) by ANOVA and Tukey's post-hoc test. (C) Kaplan–Meier survival curve with *p*=0.0035 (n=8-9; two-sided log-rank test).

Supplementary Figure 3

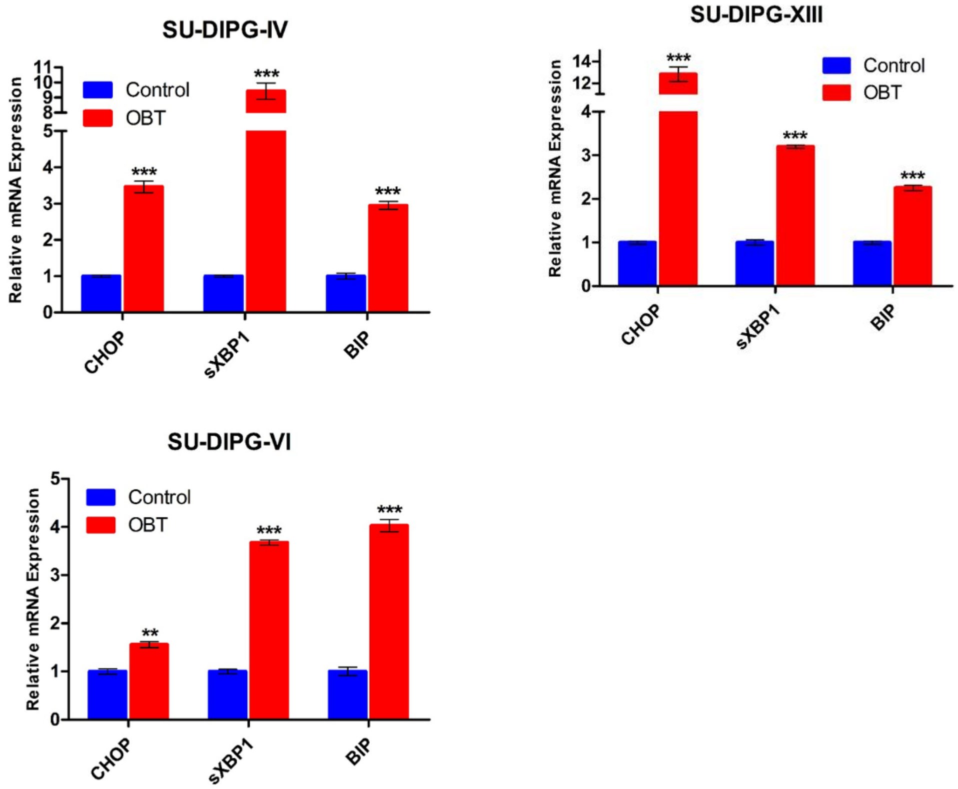

Fig. S3. SU-DIPG-IV, SU-DIPG-VI and SU-DIPG-XIII cells were treated with OBT and their mRNA were analysed by qRT-PCR for different ER stress markers including CHOP, sXBP1, and BIP. Data presented as mean ± SD; **P<0.01, ***P<0.001 vs. control by ANOVA.
